# Delayed Recognition of Acute Stroke by Emergency Department Staff Following Failure to Activate Stroke by Emergency Medical Services

**DOI:** 10.5811/westjem.2018.12.40577

**Published:** 2019-02-06

**Authors:** Joseph C. Tennyson, Sean S. Michael, Marguerite N. Youngren, Martin A. Reznek

**Affiliations:** * University of Massachusetts School of Medicine, Department of Emergency Medicine, Worcester, Massachusetts; † University of Colorado School of Medicine, Department of Emergency Medicine, Aurora, Colorado; ‡ University of Massachusetts School of Medicine, Worcester, Massachusetts

## Abstract

**Introduction:**

Early recognition and pre-notification by emergency medical services (EMS) improves the timeliness of emergency department (ED) stroke care; however, little is known regarding the effects on care should EMS providers fail to pre-notify. We sought to determine if potential stroke patients transported by EMS, but for whom EMS did not provide pre-notification, suffer delays in ED door-to-stroke-team activation (DTA) as compared to the other available cohort of patients for whom the ED is not pre-notified–those arriving by private vehicle.

**Methods:**

We queried our prospective stroke registry to identify consecutive stroke team activation patients over 12 months and retrospectively reviewed the electronic health record for each patient to validate registry data and abstract other clinical and operational data. We compared patients arriving by private vehicle to those arriving by EMS without pre-notification, and we employed a multivariable, penalized regression model to assess the probability of meeting the national DTA goal of ≤15 minutes, controlling for a variety of clinical factors.

**Results:**

Our inclusion criteria were met by 200 patients. Overall performance of the regression model was excellent (area under the curve 0.929). Arrival via EMS without pre-notification, compared to arrival by private vehicle, was associated with an adjusted risk ratio of 0.55 (95% confidence interval, 0.27–0.96) for achieving DTA ≤ 15 minutes.

**Conclusion:**

Our single-center data demonstrate that potential stroke patients arriving via EMS without pre-notification are less likely to meet the national DTA goal than patients arriving via other means. These data suggest a negative, unintended consequence of otherwise highly successful EMS efforts to improve stroke care, the root of which may be ED staff over-reliance on EMS for stroke recognition.

## INTRODUCTION

### Background and Importance

Optimizing the management of acute ischemic stroke is a priority for emergency departments (ED).^1^–^7^ Intervention for stroke patients is time sensitive, with guidelines recommending administration of intravenous (IV) recombinant tissue plasminogen activator (rt-PA) within 4.5 hours of symptom onset and initiation of endovascular procedures “as early as possible,” when indicated.^8^,^9^ Due to time sensitivity of treatment of acute stroke, emergency medical services (EMS) agencies and providers have been foci of educational efforts to drive earlier recognition. Prehospital scales have been developed to help providers identify these patients.^10^,^11^ These tools have been validated and demonstrate moderate sensitivity and high specificity.^12^–^16^ Additionally, EMS pre-notification to receiving hospitals for incoming stroke patients is considered a best practice.^4^,^13^

Multiple studies have demonstrated that pre-notification for acute stroke has improved timeliness of care including time to imaging,^1^,^3^,^4^,^7^,^12^,^13^ time to stroke team evaluation,^4^ and time to thrombolytic administration.^1^,^3^,^7^ Pre-notification also is associated with an increased rate of IV rt-PA administration overall.^4^, ^7^ While multiple investigations have shown EMS efforts in the areas of early identification and pre-notification to be successful in improving timeliness of acute stroke management, to date no research has been reported regarding potential unintended adverse consequences of these care process improvements. More specifically, we are unaware of any reports of the downstream consequences should EMS providers fail to identify an acute stroke patient and pre-notify.

### Goals of This Investigation

We postulated that EMS recognition and notification of acute stroke may be so effective that failure to pre-notify by EMS may influence timeliness of recognition of stroke symptoms by ED staff after ED arrival. We theorized that among patients for whom EMS did not recognize stroke symptoms, arrival by EMS may be associated with delays in subsequent stroke team activation by ED staff, compared to patients for whom only ED staff were responsible for recognizing stroke (i.e., those who arrived via private vehicle).

## METHODS

### Study Design and Setting

This was a cohort study in which we employed a previously developed methodology to retrospectively query the prospective stroke registry of our urban, regional referral stroke center hospital to identify all consecutive patients who presented to the adult ED and met criteria for stroke team activation between June 15, 2014, and June 15, 2015.^17^ During the study period, there were 67,795 adult ED visits and approximately 27,000 adult inpatient admissions. The center is a primary teaching site for multiple residencies, including emergency medicine (EM) and neurology, and there is a stroke team available in-house 24 hours per day, seven days a week. The ED is staffed by board-certified/board-eligible attending emergency physicians, who supervise EM and off-service rotating residents. Nursing staff are dedicated to the ED and do not float to other units, and many have achieved advanced specialty certifications. All American Heart Association/American Stroke Association *Get With The Guidelines* recommendations have been implemented,^18^ and ED nursing and physician staff undergo periodic acute stroke continuing education. We use a traditional nurse triage model.

**Population Health Research Capsule**

What do we already know about this issue?

Stroke patients for whom emergency medical services (EMS) provides prenotification to the receiving hospital experience improved timeliness of care including time to imaging and stroke activation.

What was the research question?

Do stroke patients arriving by EMS without prenotification experience the same timeliness of care as those presenting in a similarly undifferentiated state to triage?

What was the major finding of the study?

Stroke patients arriving by EMS without prenotification experience poorer timeliness of care than those who arrive to triage.

How does this improve population health?

This finding suggests the need for improved Emergency Department provider awareness of EMS patients with potential stroke as well as the need for increased awareness for EMS providers as well.

Given our geographic location and tertiary care status, the catchment area for potential stroke patients is large. Approximately 25 unique EMS agencies bring patients to our facility each year, almost all advanced life support services. EMS providers provide pre-arrival notification via radio for all inbound patients and give in-person handoff directly to ED nursing staff. EMS providers are encouraged to independently activate the stroke team from the field for patients who have a positive prehospital stroke screen. For patients without prehospital activation, ED nurses receiving in-person handoff are empowered to activate stroke resources independently, prior to physician involvement. For potential stroke patients not recognized by either EMS or ED nurses, stroke resources may be activated by a physician.

EMS providers undergo stroke recognition education as part of their biannual continuing education. In Massachusetts, EMS providers operate under standardized prehospital statewide treatment protocols, and stroke assessment is performed under the guidance of these protocols using, at the time this data were gathered, the Massachusetts Stroke Scale (MASS) or “or equivalent nationally recognized stroke scale.”^19^ The MASS is an analogue of the Cincinnati Prehospital Stroke Scale.^11^,^16^

Our investigation was approved by the University of Massachusetts Medical School Institutional Review Board.

### Selection of Participants

By protocol, the stroke team was activated when any patient presented to the ED with symptoms or findings consistent with an acute stroke within 12 hours of symptom onset. Our multidisciplinary stroke committee previously established the 12-hour window, accounting for three key considerations: prioritizing sensitivity over specificity for the mobilization of the stroke team and resources, availability of resources enabling possible treatment beyond 4.5 hours of symptoms in select cases, and institutional research protocols. The committee felt that the potential patient benefit to be gained from this expanded window outweighed the potential inefficiencies it may have caused. Because the key criteria for stroke team activation were symptoms or findings consistent with stroke at the time of activation, some patients within the registry may have had an ultimate diagnosis other than stroke, such as transient ischemic attack.

The institution maintains a prospective registry of all patients for whom the stroke team is activated, which includes patient demographics and time stamps for care events, including ED arrival, stroke team activation, computed tomography completion, and thrombolytic administration time. A stroke nurse coordinator maintains the registry and verifies its accuracy based upon established institutional guidelines. Numerous automated and manual processes exist to ensure 100% registry capture of all patients for whom stroke resources are activated.

### Methods and Measurements

Research assistants (RA), blinded to the study aims, were trained in data abstraction from the electronic health record (EHR). One study author independently abstracted at least the first 10 encounters reviewed by each RA to test for rater reliability, and there were no discrepancies. A formal analysis of inter-rater reliability was not performed. The RAs retrospectively reviewed the EHR (ED PulseCheck, Optum Clinical Solutions, Inc., Eden Prairie, Minnesota; Soarian, Cerner Corporation, North Kansas City, Missouri; and OnBase, Hyland Software, Inc., Westlake, Ohio) for each patient in the registry to validate the registry data and abstract the following fields (determined a priori) using standardized abstraction forms: mode of arrival (EMS vs non-EMS); prehospital stroke activation (yes or no); initial vital signs (heart rate, respiratory rate, systolic blood pressure, diastolic blood pressure, and pulse oxygen saturation); supplemental oxygen use and delivery method (none, nasal cannula, face mask, bag-mask ventilation, or intubated); Glasgow Coma Scale score (GCS); level of orientation (person, place, and time–range of 0–3); National Institutes of Health Stroke Scale (NIHSS) score; initial blood glucose value; elapsed time since the patient was last known to be at his or her baseline neurologic condition; previous history of stroke or transient ischemic attack; previous history of diabetes mellitus; and previous history of hypertension.

In order to identify cases in which staff inadvertently omitted documentation of prehospital activation, in the case of patients for whom there was not specific documentation regarding prehospital activation, we also compared the stroke team activation timestamp and the ED arrival timestamp. If the activation time occurred prior to the patient’s arrival, we considered prehospital activation to have occurred. Abstractors also reviewed the EHR to determine whether the ED team documented treatment of another emergent, life-threatening condition that may have delayed stroke recognition or care, such as airway/breathing intervention required, hypertension, hypotension, hypoglycemia, emergent electrolyte abnormality, or more than one of the above conditions. Rare missing values in the registry were obtained from the EHR by the abstractor.

A second investigator independently searched the EHR for missing values after the initial abstraction and also independently validated all abstracted data for a subset of cases primarily abstracted by each RA. Missing values not available in either the registry or the EHR (vital signs, n=1; glucose, n=4; and GCS, n=75) were replaced with the corresponding median value for the remaining data set, except for GCS, which was replaced with the value 15, after verifying that the remaining registry fields and NIHSS supported such as value. One entry in the registry was an exact duplicate, so the affected patient was analyzed only once. For 12 patients, there were conflicting entries between the EHR and the stroke registry as to the mode of arrival. Two senior investigators, not involved in initial abstraction (Martin Reznek and Sean Michael), reviewed each of these cases independently and blindly and had agreement upon the mode of arrival for 10 of the 12 patients (Cohen’s kappa=0.81). The two patients for whom consensus was not reached were excluded from analysis. We have previously validated and employed a similar abstraction and data verification methodology for another registry-based study.^17^

### Outcomes

Based on the electronic timestamps for ED arrival and stroke team activation, we calculated the door-to-activation (DTA) time for each patient in the stroke registry. We chose DTA to isolate any subsequent variation in stroke care processes from the process we wished to study–that of time to stroke recognition and the effect of EMS pre-notification. All subsequent stroke care processes are dependent on timely recognition of stroke syndromes and activation of stroke resources, the most appropriate measure of which is DTA. National guidelines stipulate a goal of stroke team activation within 15 minutes of patient arrival. We selected this dichotomous variable of DTA ≤ 15 minutes as our primary outcome given its prevalence in the literature as a key step in ED stoke care.^20^ Additionally, we felt it had face validity in that timely activation of resources is a prerequisite to operationalizing rapid acute stroke care, often requiring orchestration among a large and diverse team. DTA also has an inherent threshold effect on all other targets for timely stroke care in the guidelines. Achieving door-to-imaging time within 25 minutes or door-to-needle time within 60 minutes, for example, is heavily influenced by DTA (and may be impossible if DTA exceeds 25 minutes or 60 minutes, respectively).

Secondary clinical outcomes were admission to a neurology service, final ED diagnosis of stroke or intracranial hemorrhage, and administration of IV thrombolytics or neurointerventional procedure, which were recorded directly in the registry and verified in the EHR.

### Analysis

Our routine quality monitoring data suggested that approximately 72% of patients achieved DTA ≤ 15 minutes, so we estimated that the sample size required to demonstrate a two-sided difference in proportions of 10 percentage points with 80% power was 179, which was achievable using one year of registry data. We filtered the dataset to include all patients who either did not arrive via EMS or who arrived via EMS but did not have stroke team activation initiated from the prehospital setting or immediately upon arrival. Patients transferred from other facilities for stroke care were excluded, as their symptoms were, presumably, already recognized. We excluded patients with documentation of another emergent, life-threatening condition that may have delayed stroke team activation and occurred prior to activation or initial neuroimaging. Patients for whom the documented duration of symptoms was shorter than the DTA time (one possible explanation being that symptoms may have begun while already in the ED) were reviewed for potential exclusion by full-text review of the EHR documentation by a senior investigator, but no cases of documented symptom onset while in the ED were identified in the included population.

Our primary predictor of interest was mode of arrival (EMS without prehospital activation vs arrival not by EMS). We chose our comparison groups because they represent a population presenting to the ED without prior knowledge that a stroke is suspected, which allows for assessment of the time to recognition of stroke symptoms. In contrast, comparing EMS arrivals with prehospital activation to either other category risks an unbalanced comparison. We identified 17 additional candidate predictors by investigator consensus, which are listed in Tables 1a and 1b, based upon their plausibility as confounders and/or inclusion in prior studies. Preliminary analysis did not reveal a significant contribution of any temporal effects including arrival hour of day, day of week, or month/year, so we did not include any. We used a calculated mean arterial pressure (MAP) in lieu of including both systolic and diastolic values to reduce dimensionality.

Specifics of our statistical data analysis are available in web Appendix A. They are omitted from the main body of this article in the interest of brevity.

## RESULTS

### Characteristics of Study Subjects

There were 490 consecutive stroke activation patients in the registry during the study period. Of these, 383 arrived via EMS, with 277 (72.3%) presenting with EMS stroke pre-notification. Of the 213 patients who arrived either by EMS without pre-notification or arrived by other means, 11 were documented to have delays in stroke care due to a more emergent management consideration (airway/breathing intervention, n=8; hypertension, n=2; hypotension, n=1) and were excluded. NIHSS was captured on 100% of patients. Clinical outcomes of included and excluded patients are shown in the study flow diagram (Figure). Tables 1A and 1B report the baseline characteristics of included patients. The secondary clinical outcomes are similar between modes of arrival (Table 2).

Of the 200 included patients, 83 (41.5%) achieved DTA ≤ 15 minutes, and DTA ranged from < 1 minute to 3 hours 37 minutes (median 20 minutes, interquartile range [IQR] 25 minutes). Among patients who arrived via EMS without prehospital activation, 32.1% achieved DTA ≤ 15 minutes (median 22, IQR 25 minutes), compared to 52.1% among patients who did not arrive via EMS (median 14, IQR 21 minutes).

### Main Results

Overall performance of the multivariable regression model was excellent, with area under the curve 0.929. Parameter estimates for all terms are listed in Appendix A. Arrival via EMS without prehospital stroke activation, compared to arrival not via EMS, was associated with an adjusted odds ratio of 0.37 (95% confidence interval [CI], 0.15–0.92 for achieving DTA ≤ 15 minutes in the multivariable model (p=0.03). This is equivalent to a risk ratio of 0.55 (95% CI, 0.27–0.96).

## DISCUSSION

Our investigation found that potential stroke patients arriving without EMS pre-notification were only 55% as likely to meet the national 15-minute goal for DTA time as those arriving via means other than EMS. This striking finding is important and likely reflects an unintended, negative consequence of the ongoing emphasis on prehospital recognition of stroke and activation of in-hospital resources by EMS. While it is well known that pre-notification hastens ED stroke care processes, this is the first study to show that failure to pre-notify actually results in erosion of timeliness of care.

Even when controlling for demographics, patient factors (such as vital signs and history), stroke severity (including NIHSS and duration of symptoms), and propensity to arrive via EMS, stroke patients arriving via the “front door” enjoyed more timely recognition and resource activation by ED staff than those arriving via the ambulance entrance if EMS had not already recognized the stroke symptoms in the field. Our investigation was not designed to investigate causality; however, we believe the underlying mechanism is likely to be multifactorial, including triage process, ED operations (such as bed allocation), nursing assessments, or physician evaluations. Most importantly, however, we postulate that our observed results also may have been due to the success of EMS early identification and pre-notification efforts in our region, potentially creating a false sense of security and causing ED staff to become over-reliant on EMS decision making. Like our region, pre-notification success has occurred in many regions across the United States, leading us to believe that our findings may be relevant and significant for ED stroke care processes nationwide.

During the study period of our investigation, 106 patients arrived by EMS without pre-notification, while 277 (72%) did enjoy pre-notification by EMS, prompting reflection of why over a quarter of patients did not experience pre-notification by EMS. In Massachusetts, EMS providers operate under the Massachusetts Statewide Treatment Protocols, which contain and mandate the use of the MASS, or an “equivalent nationally recognized stroke scale.”^19^ This scale is analogous to the Cincinnati Prehospital Stroke Scale and therefore likely exhibits similar performance characteristics. With the Cincinnati scale, providers can be expected to demonstrate a sensitivity of approximately 60%,^11^,^16^ predicting a “miss rate” of approximately 40%. Our investigation, while not designed specifically to investigate sensitivity and specificity, found a miss rate less than 40%, in fact just slightly more than 25%. If our experience in central Massachusetts is that the screening tool is more sensitive than previously reported for the Cincinnati scale, it is possible that our proposed unintended consequence of over-reliance on EMS pre-notification may be more pronounced than in areas of the country if, and where, the sensitivity remains 40%.

The initial validation study of the Cincinnati scale listed presenting complaints of the 13 patients missed by the scale and eventually diagnosed with stroke.^16^ Among those 13 patients, seven presented with some symptom of disequilibrium such as ataxia or vertigo, and 10 were diagnosed with posterior circulation infarcts.^16^ The Cincinnati Prehospital Stroke Scale does not directly assess cerebellar function, and this may contribute to its failure to identify these patients.

Because chief complaints in our center were recorded as unstructured free-text, and total NIHSS scores do not differentiate between posterior circulation symptoms and other stroke patterns, our data provide little ability to objectively determine whether delays in recognition in our study stemmed from the same limitations. We did, however, visualize terms and phrases entered as the free-text chief complaint in our EHR among patients without pre-notification and DTA > 15 minutes using a word cloud (Appendix B). This post hoc analysis revealed a high frequency of words such as dizziness, vomiting, altered mental status, and vertigo. While this certainly cannot be used to make any firm conclusions, the similarities of our experience to the original Cincinnati investigation, in this regard, indicate that there may be potential to improve prehospital case identification with additional education for prehospital providers or even modification of the EMS stroke screening tools to better identify posterior circulation strokes. Our post hoc review, coupled with the original Cincinnati validation findings, indicate that further research may be prudent to assess the potential association between posterior circulation events and missed pre-notification.

In addition to improving prehospital identification and notification as a potential counter-measure to the primary findings of this investigation, ED provider-focused intervention may also be prudent. As EMS providers have become increasingly aware and astute at identifying strokes and providing pre-notification, it is possible that ED staff have become too reliant upon EMS identification of the patients. Additional education for ED nursing and provider staff regarding the findings of this investigation and the potential limitations of the screening tool in use by EMS may help boost index of suspicion as cases come through the door by ambulance. Further education and empowerment of triage staff may also reduce DTA times in this subpopulation.

## LIMITATIONS

The primary limitation of our investigation was the single-center design, which naturally prompts consideration of the generalizability of our results. It may be the case that our EMS and ED triage processes related to acute stroke were unique to our center and region. However, as the regional stroke center, significant efforts had been made to follow nationally accepted guidelines and recommendations in standardizing our EMS and ED stroke care, so we believe that our processes were likely to be similar to other regions and centers. As in any single-center investigation, we also could not rule out other unmeasured, locally-unique factors beyond our stroke specific processes having influenced our results. While our investigation was not multicenter, we feel that the findings remain important in that they are novel and reveal an opportunity for improvement that likely exists at centers outside of ours.

Our study intentionally did not focus on clinical endpoints, instead favoring process metrics known to affect the timeliness of stroke care. The study was not powered to evaluate door-to-thrombolytic time, given the rare nature of this outcome. Thus, it is possible that patients who experienced DTA delays fared no worse than those who met the goals; however, we feel that our primary process endpoint of DTA remains important and relevant given the accepted national emphasis on timely stroke recognition. Our results also do not precisely identify which subprocesses made the largest contributions to delays (EMS processes or care after ED arrival). Our EHR did not differentiate between arrival via basic life support (BLS) vs advanced life support (ALS). While the majority of 911-originated calls in our system arrive ALS, and both ALS and BLS providers uniformly used the MASS, it is impossible to assess for differences in prehospital stroke recognition between ALS vs BLS EMS providers.

Ambulatory patients and those arriving via EMS were initially triaged in different areas in our ED. While the triage systems were identical, there were potential differences in workflows at each of the triage areas, and it would be difficult to account for some of these factors systematically. For example, the triage nurse at our ambulance entrance had the additional responsibility of bed assignment of all incoming ED patients. While this nurse was responsible for relatively fewer patients requiring triage, it remains possible that the additional job demands may have eroded the effectiveness of stroke identification by the ambulance-entrance triage nurse. Additionally, ED patients arriving to our institution via EMS vs private vehicle, while fewer in number, were of higher acuity in general. This may have created an unmeasured demand-capacity disparity between the triage areas. However, it remains unclear in which direction this potential unmeasured effect would have biased the results, if at all. While our ED employs “pull to full” practices, and triage nurses prioritize patients using standardized scoring methods at both triage locations,^29^ during times of high ED census, ambulance patients may have had de facto priority in bed placement to facilitate EMS provider return to service. While not unique to our ED, this practice also may have created an unmeasured effect in which stroke patients not identified by the triage nurse may have had disparities in the time until their next opportunity to be evaluated by another provider, based on their arrival mode. The directionality of such an effect, however, would likely strengthen our findings, as patients arriving not via EMS would be more likely to have experienced delays.

Prehospital activation documentation was unstructured in our EHR. While it was commonly documented by providers and nurses, this left the potential that it was inadvertently omitted. We employed a secondary capture mechanism of comparing the stroke team activation time and the patient’s ED arrival time to determine whether activation had occurred for patients if prehospital activation was not specifically documented. As with any surrogate marker, there was potential that some patients may have been misclassified to either the prehospital activation or no prehospital activation group. However, we believe this to be unlikely given our objective secondary capture method. Finally, a limitation of any observational study is the possibility that unmeasured variables may play a role in confounding (i.e., influencing the probability of arrival via EMS) and/or may directly affect the outcome of interest. Our use of a prospectively-collected stroke registry with robust data-cleaning and validation somewhat mitigated this risk, as the design of the registry by multiple stakeholders and the influence of national data collection standards for accreditation were more likely to have identified important variables than the authors alone. However, our methods could not have entirely accounted for the possibility that important predictors may have gone unidentified.

Our final model’s strong area under the curve suggested against there being a large number of unmeasured predictors, but it was difficult to identify unmeasured confounders in the propensity score creation. The associated tradeoff of potential model overfitting due to the inclusion of many variables and interaction terms was also mitigated by our use of penalized regression and model validation techniques. Our final model had intuitive appeal and face validity based on theory, which also suggested against overfitting.

## CONCLUSION

In summary, our single-center data demonstrate that potential stroke patients arriving via EMS without prehospital notification (and presumably without EMS recognition of their stroke symptoms) are less likely to meet door-to-activation goals than patients arriving via other means. These results suggest the existence of a deleterious, unintended consequence of otherwise highly successful programs to improve prehospital identification and notification. The root of this unintended consequence may lie in over-reliance on EMS pre-notification and a resultant decreased index of suspicion for stroke among ED staff for patients not identified as such by EMS. Future efforts may be directed toward both increasing the sensitivity of prehospital stroke screening tools and developing improved processes for secondary screening on arrival for this cohort of patients.

## Supplementary Material





## Figures and Tables

**Figure:**
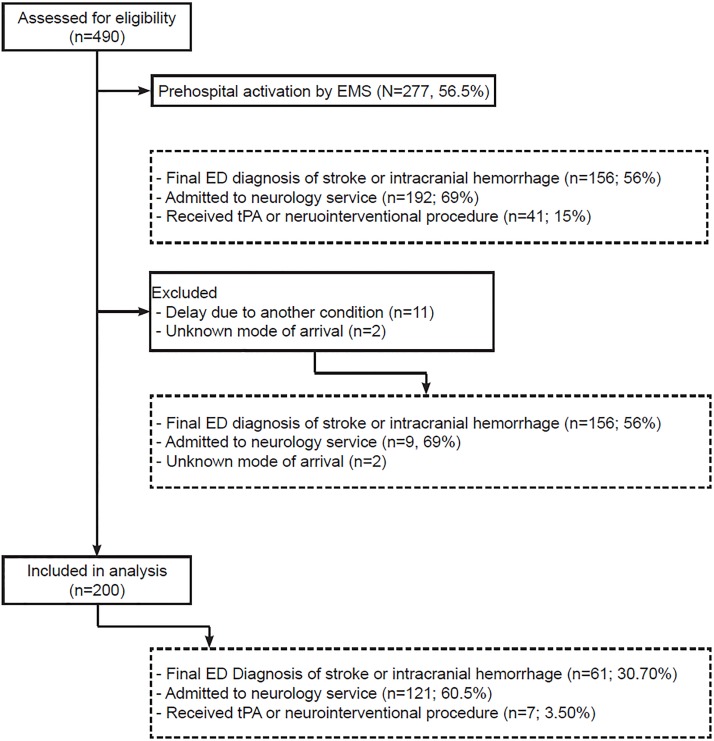
Study flow diagram. *EMS,* emergency medical services; *ED,* emergency department; *tPA,* tissue plasminogen activator.

**Table 1A:** Discrete predictor variables and study subject characteristics.

Discrete predictor	All patients n (%)	Patients arriving via EMS without prehospital activation % (n=106)	Patients arriving not via EMS % (n=94)	Patients with DTA ≤ 15 minutes % (n=83)	Patients with final ED diagnosis of stroke or ICH % (n=61)
Mode of arrival (EMS)*	106 (53.0)	100	0	41.0	50.8
Sex (female)	111 (55.5)	59.4	51.1	44.6	49.2
GCS score (<14)*	21 (10.5)	19.8	0	12.0	13.1
History of diabetes mellitus	60 (30.0)	34.9	24.5	25.3	31.1
History of hypertension	129 (64.5)	68.9	59.6	62.7	70.5
History of stroke/TIA	66 (33.0)	34.0	31.9	26.5	31.1
Orientation level (<3)*	49 (24.5)	37.9	9.6	28.9	27.9
Supplemental oxygen (intubated, high-flow, or non-rebreather mask versus nasal cannula or none)*	8 (4.0)	7.5	0	4.8	4.9

*EMS,* emergency medical services; *DTA,* door-to-activation; *ED,* emergency department; *GCS,* Glasgow Coma Scale Score; *TIA,* transient ischemic attack; *ICH,* intracranial hemorrhage.

* P-value<0.05 for univariate difference between patients arriving via EMS without prehospital activation and patients arriving not via EMS.

**Table 1B:** Continuous predictor variables and study subject characteristics.

Continuous predictor	Range for all subjects	Median (IQR) for all subjects	Median (IQR) among patients arriving via EMS without prehospital activation	Median (IQR) among patients arriving not via EMS	Median (IQR) among patients with DTA ≤ 15 minutes	Median (IQR) among patients with final ED diagnosis of stroke or ICH
Age (years)*	26–98	65 (53,76)	70 (56,82)	60 (52,70)	63 (53,76)	70 (58,78)
Blood glucose (mg/dl)	62–393	113 (98,137)	116 (101,140)	111 (97,130)	113 (99,137)	112 (98,161)
Blood pressure-systolic (mmHg)	97–232	148 (131,165)	146 (125,165)	149 (134,165)	159 (137.178)	154 (141,168)
Blood pressure-diastolic (mmHg)*	31–140	84 (73,93)	79 (68,88)	87 (78,100)	86 (71,104)	87 (75,100)
Heart rate (min-1)	37–149	79 (70,89)	80 (70,89)	79 (70,88)	83 (70,92)	79 (69,89)
NIHSS (1–42 points)*	0–25	2 (1.5)	4 (1,8)	1 (0,3)	3 (1,5)	3 (1,8)
Oxygen saturation (%)	81–100	98 (96,99)	98 (96,99)	98 (96,98)	98 (96,98)	98 (97,99)
Respiratory rate (min-1)	9–35	18 (16,20)	18 (16,20)	18 (16,20)	18 (16,20)	18 (16,20)
Time since patient last known to be at baseline neurologic condition (hours)	0.5–>12	2.5 (1.0,5.7)	2.0 (1.0,5.1)	3.0 (1.0,6.0)	2.25 (1.0,6.0)	3.0 (1.0,6.0)

*IQR,* Interquartile range; *EMS,* emergency medical services;* DTA,* door-to-activation; *ED,* emergency department; *ICH,* intracranial hemorrhage; *mg/dl,* milligrams per deciliter; *mmHg,* millimeters of mercury; *NIHSS,* National Institutes of Health Stroke Scale.

* p-value<0.05 for univariate difference between patients arriving via EMS without prehospital activation and patients arriving not via EMS.

**Table 2:** Secondary clinical outcomes by mode of arrival.

Secondary outcome	Arrival via EMS without prehospital activation (n=106)	Arrival not via EMS (n=94)	P value for difference
Final ED diagnosis of stroke or ICH n (%)	30 (28)	31 (33)	0.45
Admitted to neurology service n (%)	65 (61)	56 (60)	0.88
Received tPA or neurointerventional procedure n (%)	5 (4.7)	2 (2.1)	0.45

*EMS,* emergency medical services;* ED,* emergency department;* ICH,* intracranial hemorrhage; *tPA,* tissue plasminogen activator.
