# Supplementary material for: Delayed Recognition of Acute Stroke by Emergency Department Staff Following Failure to Activate Stroke by Emergency Medical Services
Source: West J Emerg Med. 2019 Feb 6;20(2):342–50. doi: 10.5811/westjem.2018.12.40577 (PMC6404724; doi:10.5811/westjem.2018.12.40577)
Supplement: Supplementary file 1 [file wjem-20-342-s001.docx]

**WEB APPENDIX A**

*Propensity Score*

Because of numerous confounding effects (for example, age, vital signs, past medical history, and severity and duration of symptoms may predict both arrival via EMS and DTA time), we derived a propensity score for probability of arrival via EMS using a probit regression to balance age, sex, MAP, heart rate, respiratory rate, pulse oximetry < 95%, glucose, orientation level < 3, NIHSS ≥ 2, hours of symptoms, history of diabetes, and history of hypertension, and history of stroke/TIA as predictors. Supplemental oxygen requirement more than nasal cannula and GCS < 14 exhibited near-perfect multicollinearity with arrival by EMS and were dropped as propensity score predictors. The propensity score was balanced across the exposed (arrival via EMS) and unexposed (arrival not via EMS) groups. All covariates were also balanced across mode of arrival within each quintile of the propensity score. Propensity scoring was conducted using the Stata *pscore* package (StataCorp LLC, College Station, TX).

*Regression Technique for Primary Analysis*

Given the relatively large number of predictors compared to the number of events of interest, we were hesitant to employ logistic regression alone due to the risk of overfitting. We estimated that using conventional logistic regression, our projected sample size would support a final model containing a maximum of 8 to 12 covariates, far fewer than we identified, even before accounting for interaction terms.^21^ Further explorations of potential dimensional reduction using principal components analysis did not reveal any additional opportunities to combine variables while still preserving maximal variance. However, penalized regression techniques such as least absolute shrinkage and selection operator (LASSO) have the advantage of tolerating datasets such as ours, while also performing variable selection and regularization.^22^ While less commonly published than classical techniques, this approach is well-established in the medical literature.^23-27^

We fit an adaptive LASSO regression model of DTA ≤ 15 minutes using a binomial distribution and logit link with model selection using leave-one-out cross-validation (k-fold with k=n). We considered the predictors listed previously, including two-way interactions and quadratic terms (for continuous variables) and constrained the model to force inclusion of mode of arrival and the propensity score. We converted odds ratios (OR) to risk ratios (RR) to avoid overstating effect magnitude, given the high prevalence of the outcome of interest.^28^ Analyses were conducted using JMP Pro 13 (SAS Institute Inc., Cary, NC).

As in classical logistic multivariable regression, LASSO model selection was based upon maximizing overall model performance, as opposed to including only terms significant on a univariate basis. Consequently, the final model includes some terms with parameter estimate 95% confidence intervals that cross zero or are of very small magnitude but which, nevertheless, enhance overall model prediction accuracy when included. The use of k-fold leave-one-out validation should have minimized the risk of model overfitting, and in a sensitivity analysis of the choice of validation technique, the final results for mode of arrival were insensitive to the validation method, when compared to model selection to minimize the corrected Akaike's Information Criterion (AICc).

*Secondary Analysis of DTA (as continuous independent variable)*

Despite its ubiquity in the literature, there are some limitations of using dichotomous DTA ≤ 15 minutes as a primary outcome. Consequently, we also performed a secondary analysis with DTA as a continuous independent variable. We used the same propensity score described previously and forced inclusion of the propensity score and mode of arrival in the model. We selected other dependent variables for inclusion, beginning with the same candidates as the primary analysis (without interaction effects), based upon backwards elimination to minimize AICc, using Cox proportional hazards regression with log link (because the independent variable is a time-to-event interval and violates many assumptions of ordinary least squares linear regression).

The adjusted hazard ratio (HR) for longer DTA among patients arriving via EMS without prehospital activation, compared to those arriving not via EMS, was 1.56 (95% CI 1.07 to 2.29). Other significant predictors in the continuous model were:

1. Age (HR 1.15 [95% CI 1.09 to 1.21]) per unit change
2. Heart rate (HR 0.91 [95% CI 0.86 to 0.97]) per unit change
3. Respiratory rate (HR 2.80 [95% CI 1.44 to 5.44]) per unit change
4. Glucose (HR 0.95 [95% CI 0.93 to 0.97]) per unit change
5. Propensity score (HR 362 [95% CI 47.2 to 2785]) per unit change
6. Female sex (HR 2.83 [95% CI 1.89 to 4.22])
7. Pulse oximetry <95% (HR 726 [95% CI 34.3 to 15,370])
8. Level of orientation <3 (HR 636 [95% CI 52.0 to 7793])
9. Prior stroke/TIA (HR 5752 [95% CI 166 to 199,120])
10. GCS <14 (HR 0.42 [95% CI 0.22 to 0.82])

**Web Appendix Table A**. Predictor terms included in the final LASSO regression model and their parameter estimates

| **Variable(s)** | **Specific Model Term** | **Parameter Estimate (95% CI)** | **p value** |
| --- | --- | --- | --- |
| **Single variable terms** | | | |
| Age | Age | 0.077 (0.004 to 0.151) | 0.0398 |
| Age | (Age-64.2)*(Age-64.2) | -0.001 (-0.003 to 0) | 0.0948 |
| Glucose | Initial glucose | -0.037 (-0.065 to -0.008) | 0.0114 |
| History of diabetes | Documented diabetes | 0.111 (-1.923 to 2.145) | 0.9151 |
| History of stroke/TIA | Prior documented stroke or TIA | 1.19 (-0.329 to 2.708) | 0.1247 |
| Hours of symptoms | Number of hours of sxs prior to arrival | -0.009 (-0.14 to 0.123) | 0.8993 |
| MAP | Initial MAP | -0.03 (-0.072 to 0.012) | 0.1636 |
| MAP | (Initial MAP-105.7)*(Initial MAP-105.7) | 0.001 (0 to 0.001) | 0.1662 |
| Mode of arrival | Mode of Arrival [EMS vs. Private] | -0.989 (-1.894 to -0.084) | 0.0321 |
| Propensity score | pc_pscore | 2.911 (-2.272 to 8.094) | 0.2710 |
| **Multivariable interactions** | | | |
| Age * Glucose | (Age-64.2)*(Initial glucose-129.8) | 0.001 (0 to 0.001) | 0.0476 |
| Age * History of diabetes | (Age-64.2)*Documented diabetes | -0.03 (-0.091 to 0.031) | 0.3387 |
| Age * History of stroke/TIA | (Age-64.2)*Prior documented stroke or TIA? | 0.039 (-0.006 to 0.084) | 0.0917 |
| Age * Hours of symptoms | (Age-64.2)*(Number of hours of sxs prior to arrival-3.96) | 0.004 (-0.003 to 0.011) | 0.2945 |
| Age * MAP | (Age-64.2)*(Initial MAP-105.7) | -0.002 (-0.003 to 0) | 0.0087 |
| Age * Orientation level | (Age-64.2)*Mental status is worse than 3 | -0.098 (-0.154 to -0.042) | 0.0006 |
| Glucose * Heart rate | (Initial heart rate-80.6)*(Initial glucose-129.8) | 0 (-0.001 to 0.001) | 0.8573 |
| Glucose * History of diabetes | (Initial glucose-129.8)*Documented diabetes | -0.004 (-0.023 to 0.015) | 0.6827 |
| Glucose * MAP | (Initial MAP-105.7)*(Initial glucose-129.8) | 0 (0 to 0.001) | 0.1389 |
| Glucose * Orientation level | (Initial glucose-129.8)*Mental status is worse than 3 | 0.019 (0 to 0.038) | 0.0499 |
| Glucose * Oxygen saturation | Initial pulse ox <95*(Initial glucose-129.8) | 0.029 (0.001 to 0.056) | 0.0392 |
| Glucose * Respiratory rate | (Initial respiratory rate-18.2)*(Initial glucose-129.8) | 0.001 (-0.002 to 0.004) | 0.6253 |
| Heart rate * History of diabetes | (Initial heart rate-80.6)*Documented diabetes | 0.059 (0.009 to 0.108) | 0.0197 |
| Heart rate * Hours of symptoms | (Initial heart rate-80.6)*(Number of hours of sxs prior to arrival-3.96) | 0.008 (0 to 0.016) | 0.0524 |
| Heart rate * MAP | (Initial MAP-105.7)*(Initial heart rate-80.6) | 0 (-0.001 to 0.002) | 0.8326 |
| Heart rate * Orientation level | (Initial heart rate-80.6)*Mental status is worse than 3 | 0.001 (-0.041 to 0.044) | 0.9476 |
| Heart rate * Respiratory rate | (Initial heart rate-80.6)*(Initial respiratory rate-18.2) | 0.013 (0.005 to 0.021) | 0.0019 |
| Heart rate * Sex | Sex[F vs. M]*(Initial heart rate-80.6) | -0.065 (-0.108 to -0.023) | 0.0028 |
| History of diabetes * MAP | (Initial MAP-105.7)*Documented diabetes | 0.056 (0.007 to 0.106) | 0.0260 |
| History of diabetes * Oxygen saturation | Initial pulse ox <95*Documented diabetes | 1.075 (-0.987 to 3.137) | 0.3069 |
| History of hypertension * NIHSS | NIHSS ≥2*Documented HTN | -1.758 (-3.184 to -0.332) | 0.0157 |
| History of hypertension * Oxygen saturation | Initial pulse ox <95*Documented HTN | 1.899 (0.712 to 3.087) | 0.0017 |
| History of stroke/TIA * MAP | (Initial MAP-105.7)*Prior documented stroke or TIA | 0.043 (0.007 to 0.08) | 0.0202 |
| History of stroke/TIA * NIHSS | NIHSS ≥2*Prior documented stroke or TIA? | 1.398 (-0.178 to 2.974) | 0.0821 |
| History of stroke/TIA * Orientation level | Mental status is worse than 3*Prior documented stroke or TIA? | -1.357 (-3.2 to 0.485) | 0.1488 |
| MAP * NIHSS | (Initial MAP-105.7)*NIHSS ≥2 and not missing | -0.011 (-0.051 to 0.028) | 0.5745 |
| NIHSS * Oxygen saturation | Initial pulse ox <95*NIHSS ≥2 | 0.006 (-2.123 to 2.135) | 0.9955 |
| Orientation level * NIHSS | Mental status is worse than 3*NIHSS ≥2 | -1.604 (-3.529 to 0.32) | 0.1023 |
| Orientation level * Oxygen saturation | Initial pulse ox <95*Mental status is worse than 3 | 1.67 (-0.309 to 3.648) | 0.0982 |
| Orientation level * Respiratory rate | (Initial respiratory rate-18.2)*Mental status is worse than 3 | 0.098 (-0.098 to 0.294) | 0.3253 |
| Orientation level * Sex | Sex[F vs. M]*Mental status is worse than 3 | 1.005 (-0.534 to 2.543) | 0.2007 |
| Oxygen saturation * Sex | Sex[F vs. M]*Initial pulse ox <95 | -2.483 (-3.94 to -1.026) | 0.0008 |

(Two variables joined by an asterisk indicates a model interaction term.)
