# Supplementary material for: Delayed Recognition of Acute Stroke by Emergency Department Staff Following Failure to Activate Stroke by Emergency Medical Services
Source: West J Emerg Med. 2019 Feb 6;20(2):342–50. doi: 10.5811/westjem.2018.12.40577 (PMC6404724; doi:10.5811/westjem.2018.12.40577)

Word Cloud - CC for patients who arrived by EMS, no pre-hospital activation, missed goal
Colored by probability of final diagnosis of stroke or bleed

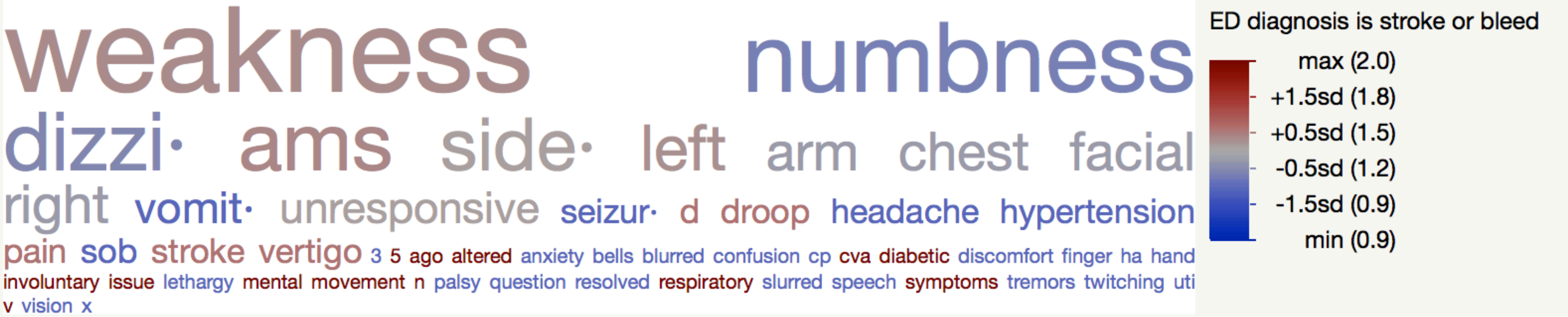

Supplement: Supplementary file 2 [file wjem-20-342-s002.pdf]
